# Skin fibroblasts from individuals with Chediak-Higashi Syndrome (CHS) exhibit hyposensitive immunogenic response

**DOI:** 10.1186/s13023-014-0212-7

**Published:** 2014-12-21

**Authors:** Le Wang, Kamila Rosamilia Kantovitz, Andrew Robert Cullinane, Francisco Humberto Nociti, Brian Lee Foster, Joseph Concepcion Roney, Anne Bich Tran, Wendy Jewell Introne, Martha Joan Somerman

**Affiliations:** NIH/NIAMS - National Institute of Arthritis and Musculoskeletal and Skin Diseases, Bethesda, MD USA; University of Campinas - Piracicaba Dental School, Piracicaba, Sao Paulo Brazil; NIH/NHGRI - National Human Genome Research Institute, Bethesda, MD USA

**Keywords:** Lysosome trafficking regulator, Intracellular vesicle trafficking, Immunodeficiency, Toll-like receptors

## Abstract

**Background:**

Chediak-Higashi Syndrome (CHS) is a rare autosomal recessive disease characterized by immunodeficiency, oculocutaneous albinism, neurological dysfunction, and early death. Individuals with CHS present with increased susceptibility to infections of the skin, upper-respiratory tract, gastrointestinal tract, and oral tissues. Classical CHS is caused by mutations in the gene encoding lysosomal trafficking regulator (*LYST*). Although defects in cytotoxic T cell lytic secretory granule secretion and neutrophil phagocytosis are suggested to contribute to the immunodeficiency in CHS, the underlying molecular mechanisms are unknown. We hypothesized that skin fibroblasts from CHS subjects exhibit impaired immune response due to defective trafficking of inflammatory factors.

**Methods and results:**

Primary skin fibroblasts from CHS subjects or healthy controls were assessed for genes encoding inflammatory response factors using PCR array. At baseline, we found *CD14, IL1R1* and *TLR-1* were down-regulated significantly (≥2 fold change) and the genes encoding *TLR-3*, *IL-1*β and *IL-6* were up-regulated in CHS cells compared to control cells. When challenged with *E. coli* lipopolysaccharide (LPS), CHS cells were less responsive than control cells, with only 8 genes significantly up-regulated (3–68 fold change) compared to baseline values, whereas 28 genes in control cells were significantly up-regulated at a much higher magnitude (3–4,629 fold change). In addition, 50% of the genes significantly up-regulated in LPS-treated control cells were significantly lower in LPS-treated CHS cells. IL-6, a fibroblast-derived proinflammatory cytokine essential for fighting infections was significantly lower in culture media of CHS cells with or without LPS. Furthermore, Western blot and immunofluorescent staining revealed that TLR-2 and TLR-4 were diminished on cell membranes of CHS cells and dissociated from Rab11a.

**Conclusions:**

For the first time, results from our study indicate defective trafficking of TLR-2 and TLR-4 contributes to the hyposensitive response of CHS skin fibroblasts to immunogenic challenge, providing a potential therapeutic target for clinical intervention in CHS.

## Background

Chediak-Higashi syndrome (CHS; OMIM# 214500) is a rare autosomal recessive disease characterized by partial oculocutaneous albinism (OCA), immunodeficiency, mild bleeding tendency, and varying neurologic problems [1,2]. Among CHS individuals, approximately 15% develop a milder form of the disease exhibiting an atypical phenotype, while the remaining 85% develop the more severe classic form of the syndrome at birth or soon after birth. It has been reported that individuals with classical CHS present persistent and recurrent infections in skin, upper-respiratory tract, gastrointestinal tract, and oral tissues [3,4]. Individuals with classical CHS often develop the “accelerated phase”, characterized by lymphoproliferative infiltration of the bone marrow and reticuloendothelial system, that is usually fatal unless treated by immunosuppression and bone marrow transplantation (BMT) [1,2,5].

The hallmark diagnostic feature of CHS is the presence of giant inclusions due to fusion of cytoplasmic granules in many cell types, including hematopoietic cells, renal tubular cells, neurons, melanocytes, and fibroblasts [1,6]. At the molecular level, the disease is caused by mutations in the lysosomal trafficking regulator gene (*LYST*, also known as *CHS1*) on 1q42.1-q42.2, identified after the murine homologue gene *beige* was discovered [7]. Studies suggest a role for LYST in vesicle formation and transport of proteins, though its dysfunction in the context of CHS is not completely understood [1,2]. Results from studies led to the suggestion that the enlarged lysosomes found in CHS cells result from abnormalities in membrane fusion [8] or fission [9], which could occur during the biogenesis of the lysosomes.

The deficiency in intracellular transport of vesicles leads to a generalized immunodeficiency in mice and humans [10,11]. Increased susceptibility to infection presented by individuals with CHS is known to be a consequence of impaired secretion of lytic secretory granules by cytotoxic T cells and defective phagocytosis, and chemotaxis by neutrophils [9,12,13]. However, other than the professional immune cells, fibroblasts, as active contributors to the regulation of the inflammatory response, provide the first barrier against pathogens [14-16]. As BMT only restores the hematopoietic stem cells but cannot correct the mutation in somatic cells such as skin and gingival fibroblasts, it is important to understand whether LYST dysfunction affects immune-inflammatory functions of fibroblasts.

Toll-like receptors (TLRs) act as essential sensors of pathogen-associated molecular patterns, ranging from lipopeptides to nucleic acids [17]. For example, *E. coli* lipopolysaccharide (LPS) bound to CD14 and MD-2 is recognized by TLR-4, controlling the expression of genes encoding several inflammatory mediators, including cyclooxygenase-2 (COX-2), and pro-inflammatory cytokines such as interleukin (IL)-1β and −6 [18]. Biological availability of TLRs has been reported to be dependent on lysosomal function, underscoring the importance of a normal lysosomal distribution for a balanced TLR response system [19]. Localization and trafficking of TLRs is essential for pathogen recognition, downstream signaling activation and modulation [19-22].

The aims of this *in vitro* study were to determine how CHS affects the immune response of skin fibroblasts and to define the mechanisms by which disturbed intracellular trafficking leads to impaired immune responses observed in individuals with CHS. We hypothesized that primary skin fibroblasts obtained from individuals with CHS would exhibit a hyposensitive response to immunogenic challenge.

## Methods

### Cell isolation, culture and treatment

A total of three subjects with classic CHS were enrolled in the Institutional Review Board approval (NIH/NHGRI - protocol #00-HG-0153) study (Table 1). Primary skin fibroblasts were obtained from these individuals with CHS. Briefly, a forearm skin biopsy was obtained under local anesthesia and enzymatically digested with 0.25% trypsin-EDTA solution (Invitrogen, CA, USA) for 1 hour at 37°C. Cells were maintained in Dulbecco's modified Eagle medium (DMEM) with 10% fetal bovine serum (FBS), 1% L-glutamine, and 1% penicillin/streptomycin (Gibco BRL) and incubated at 37°C in a 5% CO_2_ atmosphere. For the control group, cells were purchased from American Type Culture Collection (VA, USA), and stored in liquid nitrogen until use. Cells from passages 2 to 8 were used for all experiments. Twenty-four hours after plating, growth medium was changed to DMEM with 5% FBS (and penicillin, streptomycin, and L-glutamine). For baseline data, cells were cultured without *E.coli* LPS and for LPS challenge assay, cells were cultured and treated with LPS at 10 ng/mL for 3 hrs.

**Table 1:** **Genotypes of the CHS patients**

**Patients**	**Phenotype**	**Genotype**		
		**Mutation**		**Mutation type**
**CHS 4**	Classic	c.1540C > T; p.R514X (exon 5)	c.9893delT; p.F3298fsX3304 (exon 43)	Nonsense/Frameshift
**CHS 13**	Classic	c.4322_4325delAGAG;p.E1441VfsX11 (exon 12)	c.4353G > A; p.W1451X (exon12)	Frameshift/Nonsense
**CHS 21**	Classic	c.10883dupA; p.Y3628X (exon 49)	c.10883dupA; p.Y3628X (exon 49)	Nonsense/Nonsense

### Gene expression analysis

For gene expression analysis, total RNA was obtained from cells *in vitro* using the RNeasy Micro kit (Qiagen, CA, USA), cDNA was synthesized by using the RT^2^ First Strand Kit (Qiagen), and samples were analyzed for expression of 84 genes involved in immune-inflammatory regulation by a PCR array platform (PAHS-077Z, SABioscience/Qiagen). PCR array reactions were performed with the LightCycler 480 system (Roche Diagnostics, IN, USA) following the manufacturer's recommendations. Real time quantitative PCR (RT-qPCR) was performed as described before [23]. Primer sequences used in RT-qPCR are shown in Table 2.

**Table 2:** **Real-time PCR primer sequences**

**Gene**	**Accession**	**Prime forward**	**Prime reverse**
**IL-1**β	NM_000576.2	TACCTGTCCTGCGTGTGAA	TCTTTGGGTAATTTTTGGGATCT
**IL-6**	NM_000600.3	GATGAGTACAAAAGTCCTGATCCA	CTGCAGCCACTGGTTCTGT
**COX-2**	NM_000963.2	CTTCACGCATCAGTTTTTCAAG	TCACCGTAAATATGATTTAAGTCCAC
**TLR-2**	NM_003264.3	CGTTCTCTCAGGTGACTGCTC	CCTTTGGATCCTGCTTGC
**TLR-3**	NM_003265.2	AGAGTTGTCATCGAATCAAATTAAAG	AATCTTCCAATTGCGTAAAA
**TLR-4**	NM_138557.2	CCTGCGTGAGACCAGAAAG	TTCAGCTCCATGCATTGATAA
**GAPDH**	NM_002046.4	AGCCACATCGCTCAGACAC	GCCCAATACGACCAAATCC

### SDS-PAGE and Western blotting

For protein analysis, total cellular proteins were extracted from cells using a lysis and extraction kit (Thermo Scientific, MA, USA) following the manufacturer's recommendations. EDTA-free Halt™ protease inhibitor cocktail (Thermo Scientific) was included to prevent protein degradation during the extraction process. Protein concentration was determined by the Bradford colorimetric assay (Thermo Scientific).

SDS-PAGE and Western blotting were performed as described previously [23]. Primary antibodies against TLR-2 (Abcam, MA, USA), TLR-4 (Abcam), GAPDH (Abcam), and IRDye employed with secondary antibodies (LI-COR Biosciences, NE, USA) were used to detect proteins of interest. Detection was performed using a digital imaging system (ODYSSEY CLx-LI-COR), and digital files were analyzed with Image Studio Software (LI-COR).

### Immunofluorescence and membrane staining

Skin fibroblasts from both control and CHS subjects were grown on 4-well chamber slides for 24 hrs, then fixed with 4% paraformaldehyde (PFA), permeabilized using 0.1% triton-X-100, and incubated overnight at 4°C with rabbit anti-TLR-2 (Abcam), mouse anti-TLR-4 (Abcam), mouse anti-Rab11a (BD Biosciences, CA, USA), and rabbit anti-Rab11a antibodies (Cell Signaling Technology, MA, USA). Donkey anti-mouse or anti-rabbit Alexafluor-488 or −555 conjugated antibodies (Invitrogen, CA, USA) were used as secondary antibodies. The slides were mounted in ProLong Gold anti-fade reagent with DAPI (Invitrogen) and imaged using a Zeiss 510 META confocal laser-scanning microscope with the pinhole set to 1 Airy unit (Carl Zeiss, NY, USA). A series of optical sections were collected from the *xy* plane and merged into maximum projection images. For membrane staining of TLR-2 and TLR-4, cells were seeded as above and allowed to attach for 24 hrs, then cooled to 4°C and incubated for 1 hour with rabbit polyclonal antibodies against TLR-2 or mouse monoclonal antibodies against TLR-4 diluted in DMEM. Cells were washed twice for 5 minutes with ice cold PBS to remove unbound antibody, and fixed using 4% PFA for an additional 30 minutes at 4°C, and for a further 30 minutes at room temperature. The slides were then processed for immunofluorescence staining as above, using donkey anti-mouse or anti-rabbit Alexafluor-488 conjugated secondary antibodies (Invitrogen). Slides were mounted and imaged as above.

### Statistical analysis

Experiments were performed in triplicate and repeated at least twice. Values are given as means and standard deviations, or as fold-change. An integrated web-based software RT^2^ Profiler PCR array software package (SABiosciences) was used for PCR array data analysis. In general, the normality of the data is analyzed and the p values are calculated based on a Student’s *t*-test of the replicate 2 ^(−ΔCt)^ values for each gene in the control group and treatment groups. Student's *t*-test (α = 0.05) was performed for analyzing RT-qPCR data. One-way ANOVA with Tukey’s post hoc test were used for quantitation of Western blot and ELISA. Correlation analysis was performed by Pearson product moment correlation co-efficient analyses with two-tailed 95% confidence. Statistical analyses were carried out using GraphPad Prism software version 6 (GraphPad Software, CA, USA) and RT2 Profiler PCR array software package (SABiosciences).

## Results

### CHS skin fibroblasts *in vitro* exhibit hyperactive immune activity at baseline

**Figure 1:**
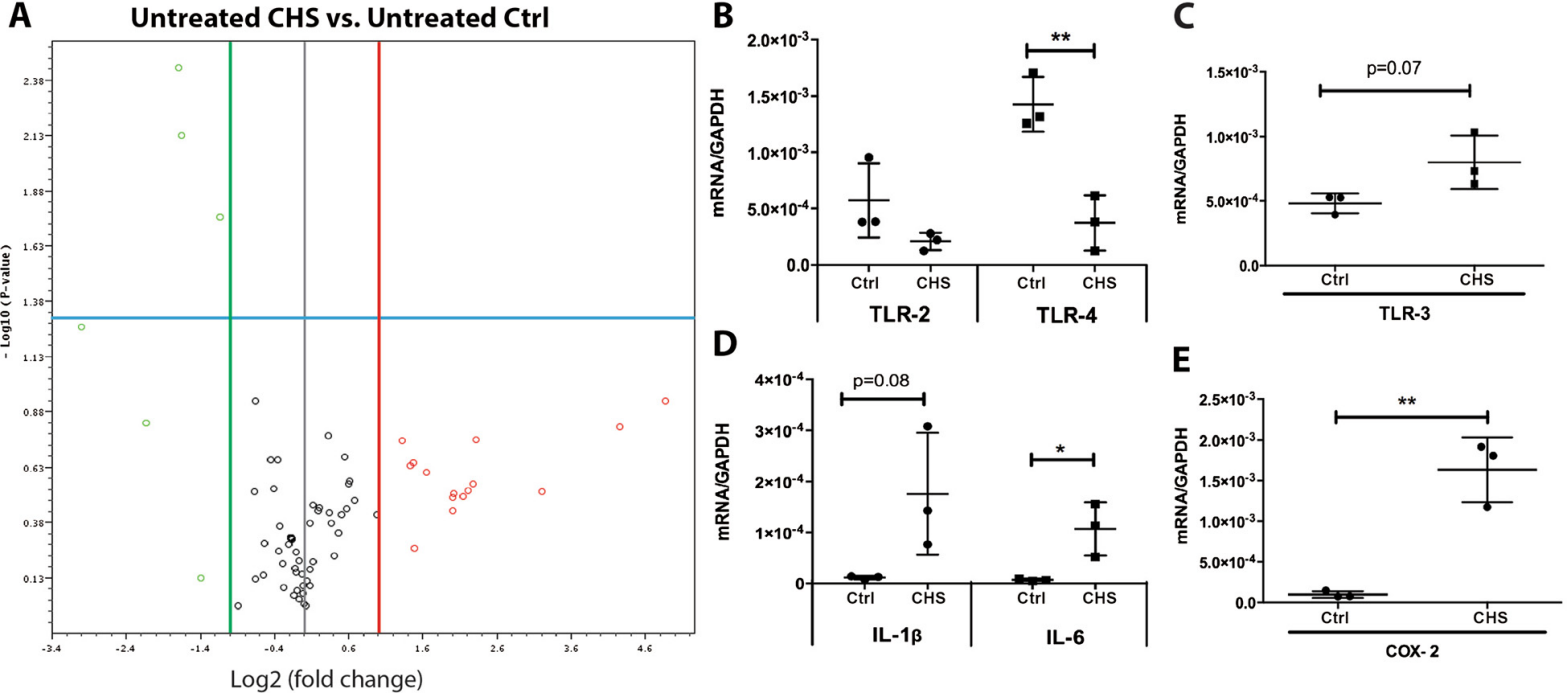
**Immune activity is hyperactive in CHS skin fibroblasts at baseline. A**. The differences in the expression of genes encoding 84 inflammation-related factors between CHS cells and control cells (Ctrl) at baseline are shown volcano plot. Y-axis shows the values of minus Log10 (p-value) and X-axis shows the values of Log2 (fold change of CHS vs. control). Red circles fall to the right of the red line represent genes increases more than 2-fold and green circles fall to the left of the green line represents genes decreases more than 2 fold. Circles above the blue line represent genes with changes of statistically significantly differences (p < 0.05 by *t*-test). Three genes, *TLR-1, IL-1R1* and *CD14* (listed in Table 3) are significantly (p < 0.05) down-regulated with 2 to 3 fold changes. RT-qPCR data reveals that compared to control cells at baseline: **B**. Expression of genes encoding *TLR-2* and −*4* are down-regulated in CHS cells at baseline; **C**. TLR-3 gene expression is enhanced in CHS cells at baseline. **D**. Expression of genes encoding *IL1*β and *IL-6* are up-regulated in CHS cells at baseline; **E**. *COX-2* gene expression is up-regulated in CHS cells at baseline. Experiments were done twice, in triplicate, with comparable results. Statistically differences were accessed by a student’s *t* test (*p < 0.05 and **p < 0.01).

To determine how CHS affects expression of genes associated with inflammation and immune response, expression profiles of skin fibroblasts at baseline were screened by PCR array and RT-qPCR. PCR array revealed that *CD14*, *IL-1R1* and *TLR-1* were significantly (p < 0.05) down-regulated, with more than a 2-fold change in CHS cells compared to control cells (Figure 1A, Table 3). Furthermore, PCR array identified decreased *TLR-2* and −*4* expression (8- and 4-fold, respectively), and 4-fold increased *TLR-3* expression in CHS skin fibroblasts compared to control cells, although without significance (Figure 1A, Table 3). RT-qPCR confirmed statistically significant 4-fold down regulation of *TLR-4* in CHS cells vs. controls (Figure 1B), while also reconfirming the expression pattern for *TLR-2* (3-fold decrease) and *TLR-3* (2-fold increase) (Figure 1B and C).

**Table 3:** **Gene expression profile of untreated CHS cells compared to untreated control cells**

**Gene symbol**	**Gene full name**	**Fold regulation**	**p-value**
**TLR1**	Toll-like receptor 1	−3.25	0.00
**CD14**	CD14 molecule	−3.14	0.01
**IL1R1**	Interleukin 1 receptor, type I	−2.19	0.02
**TLR2**	Toll-like receptor 2	−8.04	0.06
**TLR4**	Toll-like receptor 4	−4.37	0.15
**IL-6**	Interleukin 6	2.50	0.18
**IL-1**β	Interleukin 1β	9.21	0.30
**TLR-3**	Toll-like receptor 3	4.03	0.31

Note: Table 3 corresponds to the volcano plot of Figure 1A. Genes above the dotted line have altered expression in CHS cells with statistical significance (p < 0.05); Genes below the dotted line were evaluated by RT-qPCR.

RT-qPCR analysis revealed that in CHS fibroblasts, *IL-6* was significantly up-regulated (14-fold), and *IL-1*β was up-regulated (15-fold) with a p-value close to 0.05 (Figure 1D). Furthermore, expression of cyclooxygenase 2 (*COX-2*), an enzyme largely responsible for inducing inflammation, was significantly (p < 0.01) elevated (16-fold) in CHS skin fibroblasts compared to control cells. Correlation analysis shown in Table 4 indicated that expression of *COX-2*, *IL-6*, and *IL-1*β were significantly and positively correlated with one another as well as with *TLR-3* gene expression, while significantly and negatively correlated with *TLR-4* gene expression. Notably, *TLR-2* gene expression was not significantly correlated with any of the genes included in Table 4.

**Table 4:** **Summary of correlation between expression of indicated genes**

**Correlation**	**TLR-3**	**COX-2**	**IL-6**	**IL-1** **β**	**TLR-4**
**TLR-3**	n/a	r = +0.882; p < 0.05	r = +0.857; p < 0.05	r = +0.978; p < 0.001	n.s.
**COX-2**	#	n/a	n.s.	r = +0.884; p < 0.05	r = −0.911; p < 0.05
**IL-6**	#	n.s.	n/a	r = +0.829; p < 0.05	r = −0.821; p < 0.05
**IL-1** **β**	#	#	#	n/a	r = −0.831; p < 0.05

Note: **1.** TLR-2 gene expression is not significantly correlated with any of the genes shown in the table. **2.** Correlation analysis was performed by Pearson product moment correlation co-efficient analyses. Pearson r represents the correlation coefficiency where 1 is total positive correlation, 0 is no correlation, and −1 is total negative correlation. The significance is indicated by p value with two-tailed 95% confidence. n/a: not available; n.s.: not significant. #: redundant.

### CHS skin fibroblasts exhibit a hyposensitive immune response when challenged with LPS

**Figure 2:**
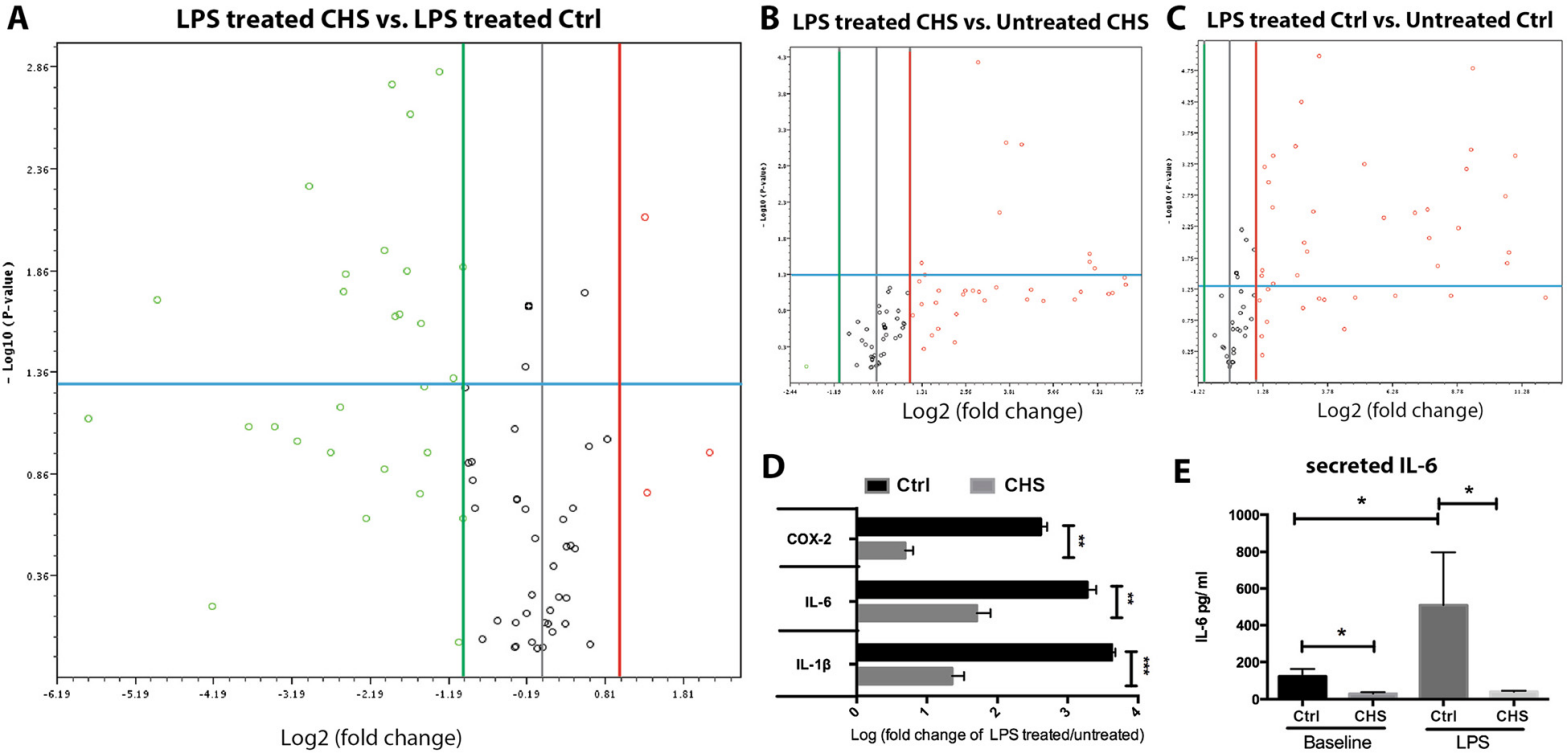
**Skin fibroblasts from individuals with CHS exhibit a hyposensitive immune response when challenged with LPS.** The differences in the expression of genes encoding 84 inflammation-related factors are shown via volcano plots **(A-C)**. Y-axis shows the values of minus Log10 (p-value) and X-axis shows the values of Log2 (Fold change of CHS vs. control). Red circles fall to the right of the red line represent genes increases more than 2-fold and green circles fall to the left of the green line represents genes decreases more than 2 fold. Circles above the blue line represent genes with changes of statistically significantly differences (p < 0.05 by *t*-test). **A**. Expression of genes encoding inflammation-related factors are repressed significantly in LPS-treated CHS cells compared to LPS-treated control (Ctrl) cells (shown in Table 5). **B**. Only 8 genes (shown in Table 6) are up-regulated significantly in LPS treated CHS cells compared to untreated CHS cells. **C**. 28 genes (shown in Table 7) are up-regulated significantly in LPS-treated control cells compared to untreated control cells. **D**. RT-qPCR data reveals that when normalized to their respective untreated cells, LPS-treated CHS cells exhibit significantly lower expression of genes encoding *IL-6, IL-1*β and *COX-2* compared to LPS-treated control cells. **E**. IL-6 expression in cell culture media evaluated by ELISA shows that IL-6 is significantly lower in culture media of CHS cells treated with or without LPS. Experiments were done twice, in triplicate, with comparable results. Statistically differences were accessed by a student’s *t* test in panel D (**p < 0.01 and ***p < 0.001) and by one-way ANOVA with Tukey’s post hoc test in panel E (*p < 0.05).

Next we examined how mutations in LYST affect inflammation and immune response genes of interest (identified in the preceding section and Figure 1) in skin fibroblasts challenged with *E. coli* LPS. The PCR array revealed that compared to LPS-treated control cells, 14 genes in CHS cells were either decreased significantly or in a declining trend (Figure 2A green circles and Table 5). This global trend indicated that, under LPS challenge, the immune response was repressed in skin fibroblasts of individuals with CHS. Notably, challenged CHS cells exhibited 30-fold lower and 4-fold lower expression of *TLR-2* and −*4*, respectively, compared to challenged control cells (Table 5). Only one gene (*LY98*) encoding *MD-2*, the LPS recognition partner of TLR-4, was significantly enhanced (2.5-fold) in CHS cells challenged by LPS (Figure 2A and Table 5).

**Table 5:** **Gene expression profile of LPS-treated CHS cells compared to LPS-treated control cells**

**Gene symbol**	**Gene full name**	**Fold regulation**	**p-value**
**TLR2**	Toll-like receptor 2	−30.20	0.02
**IL1A**	Interleukin 1, alpha	−7.85	0.01
**LTB**	Lymphotoxin beta (TNF superfamily, member 3)	−5.80	0.02
**IL23A**	Interleukin 23, alpha subunit p19	−5.67	0.01
**TLR4**	Toll-like receptor 4	−4.03	0.01
**CCL7**	Chemokine (C-C motif) ligand 7	−3.78	0.00
**CXCL3**	Chemokine (C-X-C motif) ligand 3	−3.66	0.02
**CXCR2**	Chemokine (C-X-C motif) receptor 2	−3.55	0.02
**TLR1**	Toll-like receptor 1	−3.31	0.01
**IL15**	Interleukin 15	−3.23	0.00
**CCR1**	Chemokine (C-C motif) receptor 1	−2.91	0.03
**IL18**	Interleukin 18	−2.49	0.00
**IL10**	Interleukin 10	−2.20	0.05
**IL1R1**	Interleukin 1 receptor, type I	−2.02	0.01
LY96*	**Lymphocyte antigen 96 (MD-2)**	**2.49**	**0.01**

Note: Table 5 corresponds to the volcano plot of Figure 2A. * LY96, also known as MD-2, was the only gene significantly up-regulated in LPS treated CHS cells.

The observation that CHS cells exhibited an overall lower level of immunogenic expression when compared to control cells treated by LPS does not mean that CHS cells decreased the expression of those specific factors, as shown in Table 5. By comparing stimulated cells to unstimulated cells, we revealed that both CHS and control cells exhibited increased expression of those specific factors in response to LPS challenge, but CHS cells exhibited a lower fold-response. As shown in Figure 2, only 8 genes were significantly up regulated more than 2-fold (ranging from 2.45- to 75.76-fold) in LPS-treated CHS cells compared to untreated CHS cells (Figure 2B and Table 6). In contrast, LPS-treated control cells exhibit significant up-regulation of 28 genes, more than 2-fold (ranging from 3- to 2,048-fold) compared to untreated control cells (Figure 2C and Table 7). Notably, LPS treatment significantly enhanced expression of *TLR-2* and −4 in control cells, but not in CHS cells. Consistent with trends in the PCR array data in Figure 2A, LPS-treated CHS cells featured significantly lower magnitude of *IL-1*β, *IL-6* and *COX-2* induction (40–200 fold) compared to LPS-treated control cells (Figure 2D).

**Table 6:** **Gene expression profile of LPS-treated CHS cells compared to untreated CHS cells**

**Gene symbol**	**Gene full name**	**Fold regulation**	**p-value**
**CXCL2**	Chemokine (C-X-C motif) ligand 2	75.76	0.04
**CXCL1**	Chemokine (C-X-C motif) ligand 1 (melanoma growth stimulating activity, alpha)	68.75	0.03
**IL8**	Interleukin 8	68.59	0.03
**CCL7**	Chemokine (C-C motif) ligand 7	17.88	0.00
**IL1A**	Interleukin 1, alpha	13.09	0.00
**CXCL6**	Chemokine (C-X-C motif) ligand 6	11.50	0.01
**CCL4**	Chemokine (C-C motif) ligand 4	7.53	0.00
**C3AR1**	Complement component 3a receptor 1	2.45	0.03

Note: Table 6 corresponds to the volcano plot in Figure 2B.

**Table 7:** **Gene expression profile of LPS-treated control cells compared to untreated control cells**

**Gene symbol**	**Gene full name**	**Fold regulation**	**p-value**
**CXCL3**	Chemokine (C-X-C motif) ligand 3	2048.00	0.00
**CCL5**	Chemokine (C-C motif) ligand 5	1722.16	0.01
**TNF**	Tumor necrosis factor	1652.00	0.02
**IL1B**	Interleukin 1, beta	1573.76	0.00
**IL8**	Interleukin 8	658.63	0.00
**CXCL2**	Chemokine (C-X-C motif) ligand 2	633.27	0.00
**CXCL1**	Chemokine (C-X-C motif) ligand 1 (melanoma growth stimulating activity, alpha)	557.70	0.00
**IL6**	Interleukin 6 (interferon, beta 2)	450.90	0.01
**SELE**	Selectin E	257.78	0.02
**PTGS2**	Prostaglandin-endoperoxide synthase 2 (prostaglandin G/H synthase and cyclooxygenase)	206.98	0.01
**CCL2**	Chemokine (C-C motif) ligand 2	197.18	0.00
**IL1A**	Interleukin 1, alpha	141.04	0.00
**IL23A**	Interleukin 23, alpha subunit p19	61.53	0.00
**CCL7**	Chemokine (C-C motif) ligand 7	36.50	0.00
**RIPK2**	Receptor-interacting serine-threonine kinase 2	10.78	0.00
**NFKB1**	Nuclear factor of kappa light polypeptide gene enhancer in B-cells 1	9.32	0.00
**LTB**	Lymphotoxin beta (TNF superfamily, member 3)	7.82	0.01
**CCL13**	Chemokine (C-C motif) ligand 13	7.26	0.01
**IL15**	Interleukin 15	6.74	0.00
**TLR2**	Toll-like receptor 2	6.02	0.03
**CSF1**	Colony stimulating factor 1 (macrophage)	5.76	0.00
**TLR7**	Toll-like receptor 7	3.20	0.05
**IL10**	Interleukin 10	3.20	0.00
**BCL6**	B-cell CLL/lymphoma 6	3.13	0.00
**TLR4**	Toll-like receptor 4	2.81	0.00
**CD40**	CD40 molecule, TNF receptor superfamily member 5	2.53	0.00
**IL18**	Interleukin 1	2.39	0.03
**TLR3**	Toll-like receptor 3	2.36	0.03

Note: Table 7 corresponds to the volcano plot of Figure 2C.

IL-6 is a proinflammatory cytokine that is secreted by fibroblasts [24]. Secreted IL-6 protein in tissue culture media was measured in LPS-treated and untreated cells. The concentration of IL-6 was significantly lower in medium from CHS cells compared to control cells (Figure 2E). Additionally, exposure to LPS failed to induce increased IL-6 secretion in CHS cells, while LPS-treated control cells exhibited a significant 4-fold increase in IL-6. The contradictory observation of increased IL-6 gene expression and the decreased secretion of IL-6 protein in cell culture of CHS skin fibroblasts suggests a defect in IL-6 secretion is related to LYST mutations. Notably, the difference in secreted IL-6 expression between controls and CHS fibroblasts could partly arise from the source and culturing methods of control and CHS cells. However, both control and CHS cells were cultured under identical conditions in-house and passaged several times before use in experiments, nullifying any a priori differences.

### CHS skin fibroblasts exhibit diminished protein expression of TLR-2 and −4

**Figure 3:**
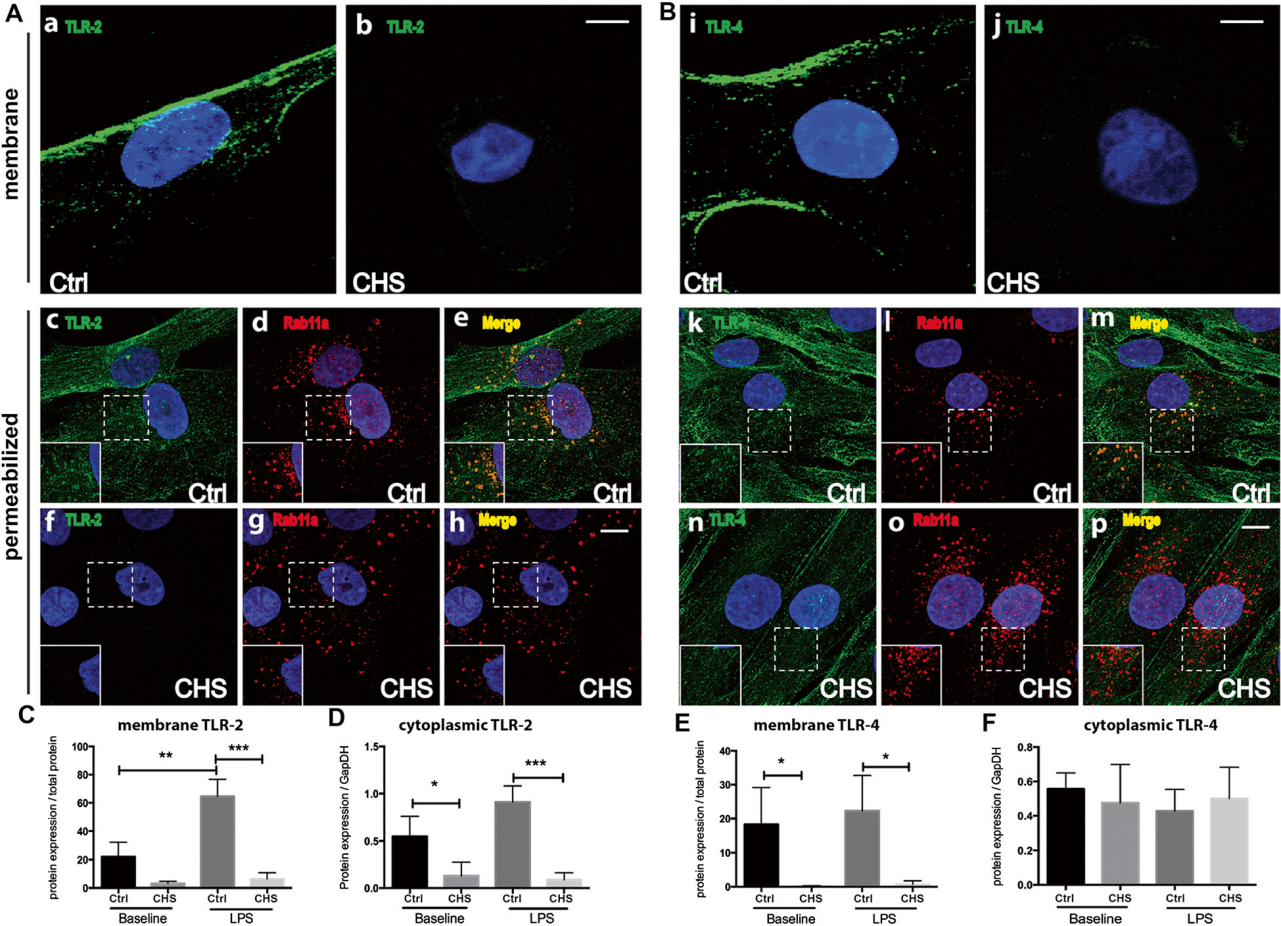
**Skin fibroblasts from individuals with CHS exhibit diminished protein expression of TLR-2 and −4. A-B.** Representative microscopy images of fibroblasts from control and a CHS patient stained for TLR-2 and TLR-4. **A**. The protein expression of TLR-2 on the plasma membrane in CHS cells is clearly reduced compared to control cells (b vs. a). Staining in permeabilized cells shows that there is still very little signal seen in CHS patient cells, suggesting that TLR-2 is being mis-trafficked and degraded in these cells (f-h). In control cells there is co-localization of TLR-2 and Rab11a shown as yellow in the merged image (c-e). **B**. Similar to TLR-2, the amount of TLR-4 on the plasma membrane in CHS cells is significantly reduced compared to control cells (j vs. i). Staining in permeabilized cells shows that there is a noticeable reduction in signal in CHS cells (n). In control cells there is co-localization of TLR-4 and Rab11a (m), but very little co-localization can be seen in CHS cells (p). Inserts are enlarged images of area inside dashed boxes. Scale bar represents 20 mm. **C-F**. Quantitative analysis of Western blot result shows the expression of TLR-4 and −2 of cells, treated with or without LPS. Experiments were done twice, in triplicate, with comparable results. Statistically differences were accessed by one-way ANOVA with Tukey’s post hoc test in panel E (*p < 0.05, **p < 0.01 and ***p < 0.001).

Toll-like receptors are recognized as playing key roles in innate immune response to immunogenic challenges, such as microbial products including LPS [17]. Lower gene expression of *TLR-2* and *TLR-4* in CHS fibroblasts compared to controls cells (Figure 1A and B) prompted us to analyze protein expression and localization to further define the mechanism for the observed defective immune response in CHS cells. At baseline, membrane-bound TLR-2 and −4 were decreased markedly in CHS versus control cells (Figure 3A-a, b and 3B-i, j), with significant differences confirmed by quantitative analysis of Western blot data (Figure 3C and E).

Cells were permeabilized to visualize cytosolic TLR-2 and −4, revealing that compared to robust cytosolic TLR-2 in control fibroblasts, cytosolic TLR-2 was not detectable in CHS cells (Figure 3A-f vs. c). Quantitative analysis of Western blot confirmed this observation (Figure 3D). In contrast, cytosolic TLR-4 was comparable in CHS versus control cells (Figure 3B-n vs. k), and Western blot analysis confirmed this observation (Figure 3F). The Rab11a GTPase has been shown to be involved in the recycling and trafficking of TLRs [20]; therefore we co-stained Rab11a with TLR-2 and −4. In control fibroblasts, Rab11a co-localized with TLR-2 and −4 (Figure 3, A-e and B-m). However, in CHS cells, Rab11a localization was notable for a lack of co-localization with TLR-4 (Figure 3B-p). Furthermore, Western blot revealed that when challenged by LPS, CHS cells exhibited significantly lower expression of TLR-2 compared to control cells (Figure 3C and D). Although intracellular TLR-4 expression in CHS cells was similar to that of control cells, the expression of membrane bound TLR-4 was significantly decreased in CHS cells (Figure 3E and F).

## Discussion

Individuals with CHS are reported to exhibit increased susceptibility to infections in tissues that act as barriers to pathogenic invasion, such as the skin and gingiva [3,4]. Fibroblasts, the major cells residing in skin and gingiva, are immune competent cells that participate in signaling and triggering an innate immunity reaction in response to pathogens [24,25]. While CHS-induced defects have been studied in major immune cell classes, including neutrophils [26,27], macrophages [28], and T cells [29], little is known about how mutations causing CHS affect immunogenic responsiveness of fibroblasts. In these *in vitro* studies of skin fibroblasts obtained from individuals with CHS, we demonstrate hyperactive inflammatory immune activity at baseline, yet hyporesponsiveness to immunogenic LPS challenge, potentially due to the reduced expression and disturbed trafficking of TLR-2 and −4.

Our results demonstrate that at baseline (i.e. unstimulated), CHS fibroblasts present hyperactive expression of immune-related factors including *IL-1*β, *IL-6*, and *COX-2*, compared to cells obtained from normal subjects. Enhanced expression of these factors was significantly and positively correlated with one another and with enhanced *TLR-3* expression in CHS cells, suggesting co-regulation by common upstream signals. In fact, TLR-3 is one of the upstream regulators that has been shown to induce expression of the elevated immune-related factors mentioned above (Figure 1D) [30]. Although *TLR-1, −2, −4*, and *CD14* were all decreased in CHS cells, increased expression of *TLR-3* may lead to the mild hyperactive immune activity. The marked functional difference between TLR-2 or −4, and TLR-3, is that TLR-2 and −4 localize to the cell surface in order to recognize foreign lipid structures, and rely on intracellular trafficking to signal and replenish the surface pool of TLR proteins [21], while TLR-3 resides intracellularly in order to recognize nucleic acids delivered by a cytoplasmic lipid raft protein, reftline, and depends on intracellular trafficking differently from TLR-2 and −4 [31,32]. LYST protein, as an intracellular trafficking regulator whose function is reduced in individuals with CHS, may have a more potent effect on highly regulated cell surface expressed TLRs (e.g. TLR-2 and −4), than intracellular and constitutive TLRs (e.g. TLR-3). It is plausible to propose that skin fibroblasts constantly exposed to exogenous viruses exhibit enhanced expression of *TLR-3* and its downstream signaling, which is affected differently by LYST mutation compared to cell surface TLRs and in turn leads to a hyperactive immune system at baseline.

In contrast to the hyperactive expression at baseline, skin fibroblasts from CHS individuals exhibit marked hyporesponsiveness to LPS challenge, failing to alter production of chemokines and cytokines including *CCL-7, IL-10, IL-15, IL-18, IL-1A*, and *IL-23A*. Repeated activation of TLR signaling results in a reduction in the subsequent proinflammatory cytokine response, a phenomenon known as TLR tolerance [33], as well as changes in expression of other TLRs, known as cross-tolerance [34]. In the case of CHS cells, gene expression and correlation data suggest that constant activation TLR-3 signaling potentially leads to elevated expression of cytokines and chemokines, which in turn represses the expression of *TLR-4* and results in hyposensitive immunogenic response. However, it remains unclear why changes in *TLR-2* did not correlate with increased expression of other factors. TLR2 signaling induction relies on heterodimerization with TLR-1 or TLR-6 [35-37]. Significantly diminished expression of *TLR-1* in CHS cells may, explain in part, the complexity of the altered TLR-2 expression.

Cytoplasmic membrane-associated TLRs signal through two primary pathways, defined by the adaptor molecules used to initiate each signal cascade. The classical MyD88-dependent pathway relies on functioning cell surface TLRs, is common to both TLR-2 and −4, and leads to rapid activation of transcription factor κB (NF-κB) to induce proinflammatory mediators such as TNF-α, IL-6 and COX-2 [17]. Our results support severely deficient MyD88-dependent TLR signaling in CHS cells, based on the observations of (1) decreased gene expression of *TLR-2, −4,* and *CD14*; (2) attenuated cell surface expression of TLR-2 and −4; and (3) diminished response under LPS challenge of MyD88-dependent proinflammatory mediators, e.g. IL-6 and CXCL2. Furthermore, the presence of TLR-4 receptor complexes on the cytoplasmic membrane is maintained via continuous replenishment of TLR-4 from intracellular compartments including the Golgi apparatus and endosomes, which is a process that is governed by small GTPase and MD-2 [22,38]. CHS skin fibroblasts exhibited enhanced expression of *MD-2*, suggesting a compensatory mechanism that minimizes alterations in cytoplasmic TLR-4 protein in the face of reduced *TLR-4* expression. It will be important to investigate how LYST affects interactions between TLR4 and MD-2, in order to modulate cell surface localization of TLR-4.

In addition to MyD88-dependent TLR signaling from the cell membrane, internalization of TLRs can facilitate a second signaling pathway that employs a distinct set of sorting-signaling adaptors, called the MyD88-independent pathway [17]. Although TLR-2 and −4 share some of the MyD88-independent pathway adaptors, signaling activation of this pathway has different consequences for intracellular trafficking of TLR-2 versus TLR-4. Internalization of TLR-4, governed by the small GTPase, Rab11a, is necessary to induce MyD88-independent signaling [20] while internalization of TLR-2 is not necessary for signaling [39]. This difference may underlie the discrepancy in how LYST mutations affect TLR-2 and TLR-4 in CHS cells. In addition, internalization also results in the TLR recycling through ubiquitination [21]. Since TLR-2 and TLR-4 differ in their mechanism of ubiquitination [40,41], incorrect trafficking may lead to severe depletion of TLR-2, but not TLR-4, in CHS cells. Future studies elucidating this mechanism will provide insight into how LYST contributes to TLR-2 and −4 functions and recycling.

To the best of our knowledge, the results reported here demonstrate for the first time the altered expression and localization of TLRs in cells obtained from CHS subjects, and emphasize the importance for understanding the mechanism by which lysosome recycling regulates TLR-mediated inflammatory signaling, and immune function in a broader sense. Clinical features resulting from *LYST* mutations in CHS have much in common with immunodeficiencies caused by TLR signaling defects, such as conditions caused by autosomal recessive mutations in TLR adapters, *IRAK-4* and *MyD88* (OMIM# 610799, 607676, 612260). Like mutations in *LYST*, reduced function of IRAK-4 and MyD88 results in selective impairment of cell responsiveness to TLRs other than TLR-3 [42], and limited presence of IL-6 protein when exposed to TLR agonists [43]. These conditions feature noninvasive pyogenic bacterial infections affecting skin and upper respiratory tract, with occasional periodontal disease [43]. However, patients with MyD-88 and IRAK-4 deficiency show no impaired defense against viral infections [43], due to their normal functional natural killer cells [43] as well as their retained ability to signal through TLR-3/-7/-9 and other non-TLR viral receptors [44]. In contrast, patients with the classical CHS phenotype develop life-threatening haemophagocytic lymphohisticytosis following infections with viruses [5], which may result from dysfunctional natural killer cells lacking cytotoxic activities [45,46] as well as defective trafficking in TLR-3/-7/-9 signaling.

## Conclusions

Taken together, these findings underscore that intracellular vesicle trafficking is essential for normal immune function. Loss of expression or proper localization of TLR-2 and −4, together with the lack of response of cell production of pro-inflammatory cytokines, leads to exacerbated bacterial burden and delayed clearance. A better understanding of mechanisms governing local inflammatory mechanisms may inform strategies for the management of skin lesions burdened by excessive inflammation, in CHS and other conditions of immunodeficiency.

## Abbreviations

BCL6: B-cell CLL/lymphoma 6
BMT: Bone marrow transplant
C3AR1: Complement component 3a receptor 1
CCL: Chemokine (C-C motif) ligand
CCR: Chemokine (C-C motif) receptor
CD14: Cluster of differentiation 14
CEBPB: CCAAT/enhancer binding protein (C/EBP), beta
CHS: Chediak-Higashi syndrome
Ctrl: Control
COX-2: Cyclooxygenase-2
CSF1: Colony stimulating factor 1 (macrophage) CXCL, Chemokine (C-X-C motif) ligand
CXCR: Chemokine (C-X-C motif) receptor
DMEM: Dulbecco modified Eagle medium
FBS: Fetal bovine serum
IL: Interleukin
IRAK: Interleukin-1 receptor-associated kinase
LPS: Lipopolysaccharide
LTB: Lymphotoxin beta (TNF superfamily, member 3)
LY96: Lymphocyte antigen 96, also known as MD-2
LYST: Lysosomal trafficking regulator
MD-2: Myeloid differentiation factor-2
MyD-88: Myeloid differentiation primary response gene 88
NFKB1: Nuclear factor of kappa light polypeptide gene enhancer in B-cells 1
OCA: Oculocutaneous albinism
PFA: Paraformaldehyde
Rab: Ras superfamily of monomeric G proteins
RIPK2: Receptor-interacting serine-threonine kinase 2
SDS-PAGE: Sodium dodecyl sulfate-Polyacrylamide gel electrophoresis
SELE: Selectin E
TLR: Toll-like receptor
TNF: Tumor necrosis factor
